# Hydrosilylation Reactions Catalyzed by Rhenium

**DOI:** 10.3390/molecules26092598

**Published:** 2021-04-29

**Authors:** Duo Wei, Ruqaya Buhaibeh, Yves Canac, Jean-Baptiste Sortais

**Affiliations:** 1 University Rennes, CNRS, ISCR-UMR 6226, 35000 Rennes, France; Duo.Wei@catalysis.de; 2 LCC-CNRS, Université de Toulouse, UPS, 31400 Toulouse, France; ruqaya.buhaibeh@lcc-toulouse.fr (R.B.); yves.canac@lcc-toulouse.fr (Y.C.); 3 Institut Universitaire de France 1 rue Descartes, CEDEX 05, 75231 Paris, France

**Keywords:** rhenium, hydrosilylation, unsaturated bonds, carbonyls, alkenes

## Abstract

Hydrosilylation is an important process, not only in the silicon industry to produce silicon polymers, but also in fine chemistry. In this review, the development of rhenium-based catalysts for the hydrosilylation of unsaturated bonds in carbonyl-, cyano-, nitro-, carboxylic acid derivatives and alkenes is summarized. Mechanisms of rhenium-catalyzed hydrosilylation are discussed.

## 1. Introduction

**Figure 1:**
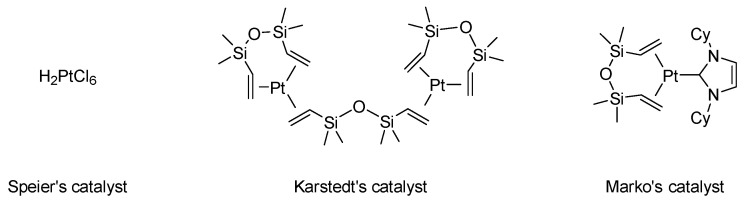
Representative platinum-based hydrosilylation catalysts.

Hydrosilylation is a very versatile transformation consisting of the addition of a hydrosilane (H-SiR_3_) to an unsaturated bond. Organosilicon compounds have found widespread applications in our daily lives in silicon-based materials such as silicon rubbers, adhesives, paper release coating, and so forth. In addition, hydrosilylation is an atom economic reaction to access valuable organosilane intermediates for fine chemical synthesis [1,2]. For many years, platinum has been the metal of choice for designing hydrosilylation catalysts, Speier’s, Karstedt’s or Markó’s catalysts being the most representative examples (Figure 1). Other noble transition metals, such as rhodium, ruthenium, iridium or palladium, have also been used in this transformation [3]. However, the limited availability of these precious transition metals has prompted researchers to explore alternatives metals in the periodic table, in particular first row transition metals such as iron, cobalt and nickel [4,5,6].

In this regard, group 7 transition metals, manganese [7] and rhenium [8], were not an obvious choice at first glance. Indeed, rhenium complexes, in particular oxo-rhenium compounds, have been mainly recognized as efficient catalysts in oxidations, such as epoxidation or oxygen atom transfer reactions [9,10,11,12,13,14]. This is highlighted by organo-rhenium(VII) trioxide, particularly methyltrioxorhenium (MeReO_3_, abbreviated as MTO), arguably one of the most versatile transition metal catalysts known to date. Rhenium is also well-known to promote olefin metathesis [15] or aldehyde olefinations [16]. In complement, the coordination chemistry of rhenium was explored for the potential application of ^186/188^Re radioisotopes in nuclear medicine and bio-medical chemistry [17,18].

The application of rhenium derivatives in hydrosilylation [19,20] was not unveiled until 2003 [21,22]. Since the application of the lighter congener of rhenium, namely manganese, has been increasing lately in hydrosilylation [23,24], we report here the evolution of rhenium in this catalytic transformation.

## 2. Hydrosilylation of C=O, C=N, C≡N and NO_2_ Bonds

### 2.1. Hydrosilylation of Carbonyl Derivatives

**Scheme 1:**
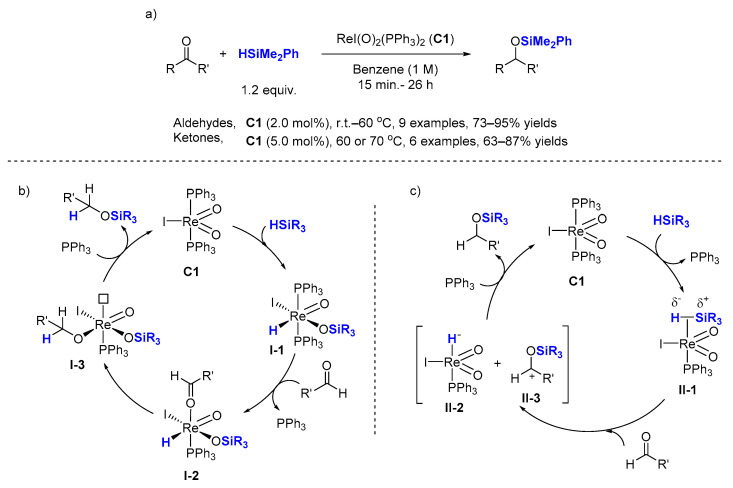
ReI(O)_2_(PPh_3_)_2_ (**C1**) catalyzed hydrosilylation of carbonyl compounds with related mechanism studies. (**a**) Scope of the reaction; (**b**) Proposed [2 + 2] addition mechanistic pathway; (**c**) Proposed ionic outer-sphere mechanistic pathway.

The first rhenium-catalyzed hydrosilylation was described in 2003 by Toste et al. (Scheme 1a). This seminal contribution represents the first example of a hydrosilylation catalyst with a high-valent metal bearing two terminal oxo ligands, reversing the traditional use of metal-oxo complexes in catalysis. Indeed, the hydrosilylation of aldehydes and ketones was promoted by the readily available iododioxo(bistriphenylphosphine)rhenium(V) complex [(PPh_3_)_2_Re(O)_2_I] (**C1**) [21,22]. The scope of this reaction included aromatic or aliphatic ketones and aldehydes with a good tolerance of functional groups (amino, nitro, halo, ester, cyano, cyclopropyl and alkene groups remained untouched). This air and moisture tolerant reaction provides silyl-protected alcohols in a straightforward reduction-protection protocol as bulky hydrosilanes were used (HSiMe_2_Ph, HSiEt_3_, HSiMe_2_Ph and HSitBuMe_2_).

A detailed mechanism, supported by experimental evidence, was proposed by the same group [21,25] and confirmed by a computational study by Wu et al. in 2006 [26] (Scheme 1b). The first step involves the formal [2 + 2] addition of silane to the Re=O bond in **C1** to produce metal hydride **I-1**, which was isolated and characterized by X-ray diffraction. Alkoxy-metal intermediate **I-3** is produced by the addition of rhenium hydride to the carbonyl group. Transfer of the silyl group to the alkoxy ligand, formally a *retro*-[2 + 2] reaction, forms the silyl ether product and regenerates the dioxo catalyst **C1**.

However, the computational study by Wei [27] showed that the ionic outer-sphere mechanistic pathway (Scheme 1c) is energetically more favorable than the [2 + 2] addition mechanism supported by Wu for the **C1**. In this alternative mechanism, the activation of Si−H goes through η^1^-bonding of silane to the metal center followed by a nucleophilic addition of the organic substrate on the Si center in **II-1** to cleave the Si−H bond forming the anionic rhenium hydride **II-2** and the activated organic substrate **II-3**. Finally, hydride transfer from the rhenium metal **II-2** to the carbon atom of the organic substrate **II-3** produces the desired product and regenerates complex **C1**.

**Scheme 2:**
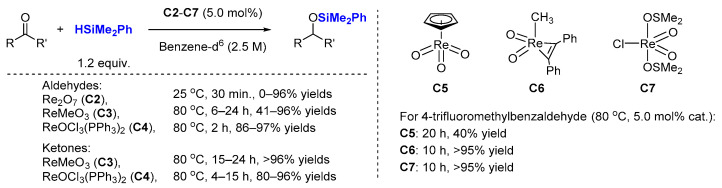
Reduction of carbonyl groups by high-valent rhenium oxides **C2**–**C7**.

Intrigued by the work of Toste, Royo et al. explored in 2005 the catalytic activity of a family of oxo-rhenium complexes (**C2**–**C7**, Scheme 2) [28]. All these complexes showed activity in the reduction of aliphatic and aromatic aldehydes (six examples) and ketones (four examples) with dimethylphenylsilane in C_6_D_6_ solution (Scheme 2), demonstrating the general ability of oxo-rhenium complexes to promote hydrosilylation. Notably, **C2** catalyzes the hydrosilylation of aldehydes at room temperature, within 30 min, affording the corresponding silyl ethers in a good yield, but is ineffective as a ketone hydrosilylation catalyst (even at 80 °C). **C5**–**C7** (5.0 mol%) are active for the hydrosilylation of 4-trifluoromethylbenzaldehyde at 80 °C, giving 40% yield (**C5**, 20 h), >95% yield (**C6**, 10 h) and >95% yield (**C7**, 10 h), respectively.

**Scheme 3:**
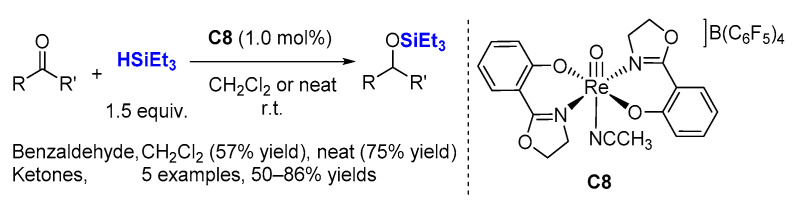
Reduction of carbonyl groups by oxazoline containing rhenium oxide **C8**.

In the same year, Abu-Omar and co-workers reported a new system for the hydrosilylation of aldehydes and ketones using a mono-oxorhenium(V) catalyst (**C8**) containing two oxazoline ligands (Scheme 3) [29,30]. The reaction proceeds efficiently under ambient temperatures with low catalyst loading (0.1 mol%), 1.5 equiv. HSiEt_3_ using CH_2_Cl_2_ as a solvent or without a solvent. Interestingly, the catalyst preserves its activity after being recycled (for 2-butanone, 80% NMR yield (1st cycle) and 50% NMR yield (2nd cycle)).

**Scheme 4:**
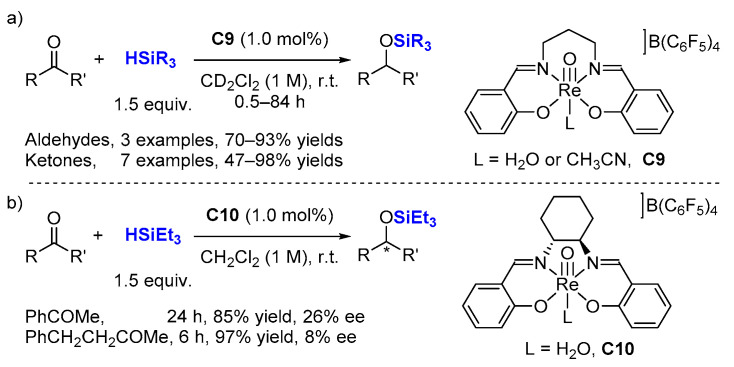
Hydrosilylation of carbonyl compounds with cationic oxorhenium(V) non chiral salen (**a**) and chiral salen (**b**) based complexes.

Then, Abu-Omar and co-workers prepared a number of cationic oxorhenium(V) salen based complexes such as [Re(O)(salpn)(Solv)][B(C_6_F_5_)_4_] **C9** [31,32] in 2006 (Scheme 4a) and [ReO(saldach)(H_2_O)][B(C_6_F_5_)_4_] **C10** [33] in 2008 incorporating a chiral environment, (Scheme 4b). **C9** and **C10** serve as good catalysts for hydrosilylation of carbonyl compounds (1.0 mol% cat. loading, 1.5 equiv. silane, room temperature (r.t.), although asymmetric versions (**C10**) of these reactions afford poor enantioselectivity, even in the presence of bulky silane such as HSitBuMe_2_.

**Scheme 5:**
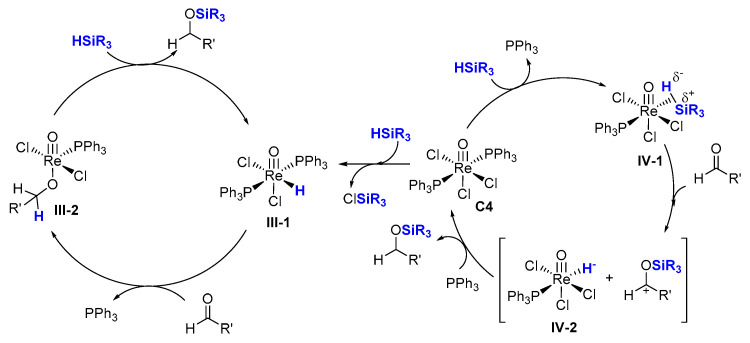
The σ-bond metathesis pathway involving the isolated hydride intermediate Re(O)Cl_2_H(PPh_3_)_2_ (**left**). The pathway involving a η^2^–silane complex (**right**).

The mechanism of action of these high-valent mono-oxo-rhenium(V) catalysts was investigated, providing new insights [34] including via a theoretical study [35,36,37]. The addition of silane across the Re-O multiple bond was not observed in mono-oxo-rhenium(V) complex Re(O)Cl_3_(PPh_3_)_2_ (**C4**) catalyzing the hydrosilylation of carbonyls. Furthermore, they observed that silanes slowly reacted with **C4** to give the isolated hydride intermediate Re(O)Cl_2_H(PPh_3_)_2_ (**III-1**). However, this isolated hydride intermediate is not sufficiently reactive to account for the catalytic turnover, as only 20% yield of the silyl ether product is observed (Scheme 5 left). These observations lead the authors to propose that the reaction pathway involves the formation of a η^2^–silane complex (**IV-1**), followed by the heterolytic cleavage of the Si-H bond at the rhenium center after elimination of one PPh_3_ ligand (Scheme 5, right). This ionic mechanism involves the sequential transfer of a silyl ion (R_3_Si^+^) and then a hydride to a polar double bond to furnish the reduction reactions. In this proposed catalytic cycle, neither the coordination of unsaturated substrates to the metal center nor its insertion into the M-H bond is involved.

**Scheme 6:**
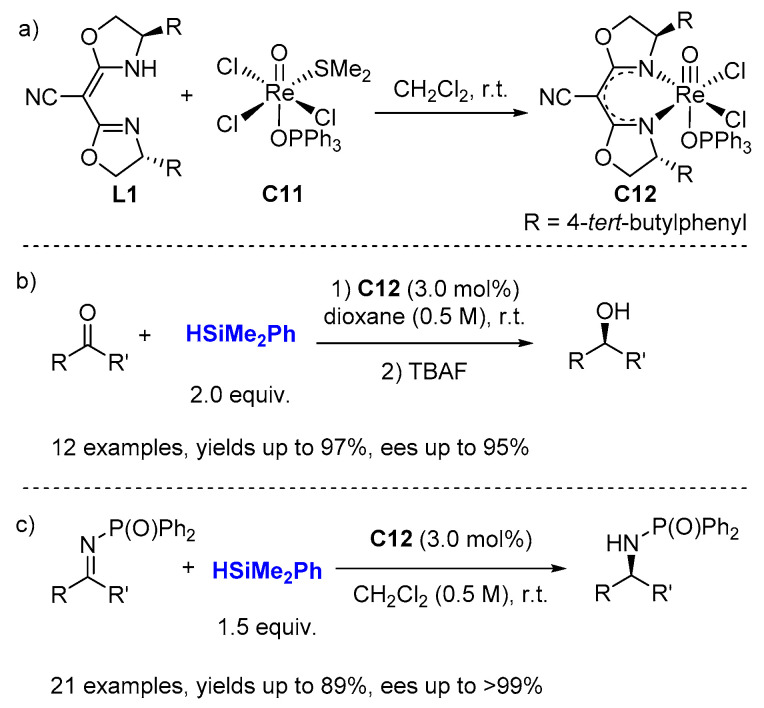
Enantioselective reduction of ketones (**b**) and imines (**c**) catalyzed by (CN-Box)Re(V)–oxo complexes **C12** (**a**).

The enantioselective reduction of prochiral ketones [38] and imines [39] with 2 equiv. HSiMe_2_Ph was reported by Toste (Scheme 6) using chiral (CN-box)Re(V)–oxo complexes (3.0 mol%, CN-Box = cyanobis(oxazoline)). These reduction reactions proceed under an ambient air atmosphere with a highly functional group tolerant with ees of up to >99%. The (CNbox)Re(V)–oxo complexes (**C12**) can be prepared by simply stirring **L1** with Re(O)Cl_3_(OPPh_3_)(SMe_2_) (**C11**) in CH_2_Cl_2_ at r.t. and isolated as a green stable solid (Scheme 6a) or generated directly in situ.

Similarly, a theoretical investigation of the mechanism for the high-valent mono-oxo-rhenium(V) hydride Re(O)HCl_2_(PPh_3_)_2_ (**III-1**) catalyzed hydrosilylation of C=N functionalities was performed by Wei and co-workers [40]. These results suggest that an ionic S_N_2-Si outer-sphere pathway proceeding via the heterolytic cleavage of the Si-H bond competes with the hydride pathway featuring the C=N bond inserted into the Re-H bond.

**Scheme 7:**
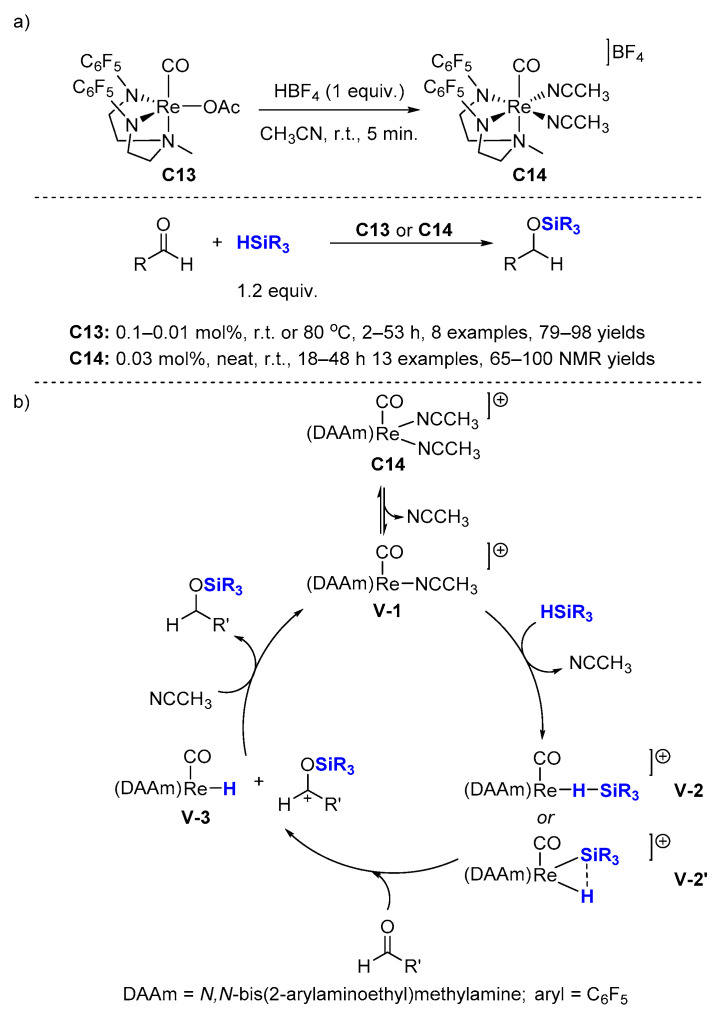
Cationic rhenium complex catalyzed hydrosilylation of aldehydes. (**a**) Scope of the reaction; (**b**) Proposed mechanism.

Besides these oxorhenium complexes, a series of novel low-valent Re(III) complexes were synthesized by Ison et al., such as **C13** [41] in 2012 and **C14** [42] in 2017 (Scheme 7a). **C14** (0.03 mol%, r.t.) is more reactive than **C13** (0.1 mol%, 80 °C) for the hydrosilylation of aldehydes [42]. Excellent NMR yields (65–100%, 13 examples) were achieved at ambient temperature under neat conditions using dimethylphenylsilane with **C14**. The reaction affords TONs of up to 9200 and a TOF of up to 126 h^−1^ (Scheme 7a).

Based on kinetic and mechanistic studies, an ionic outer-sphere mechanism for the catalytic hydrosilylation of benzaldehyde by **C14** has been proposed (Scheme 7b). First, silane is activated through η^1^ (**V-2**) or η^2^ (**V-2′**) coordination to the rhenium center. Then, the aldehyde substrate attacks the Si center in (**V-2**) or (**V-2′**) to prompt the heterolytic cleavage of the Si-H bond yielding an ion pair, the hydride complex **V-3** and a silylated aldehyde ion. Finally, hydride transfer from rhenium to the carbonyl carbon of the activated substrate completes the catalytic cycle [43].

**Scheme 8:**
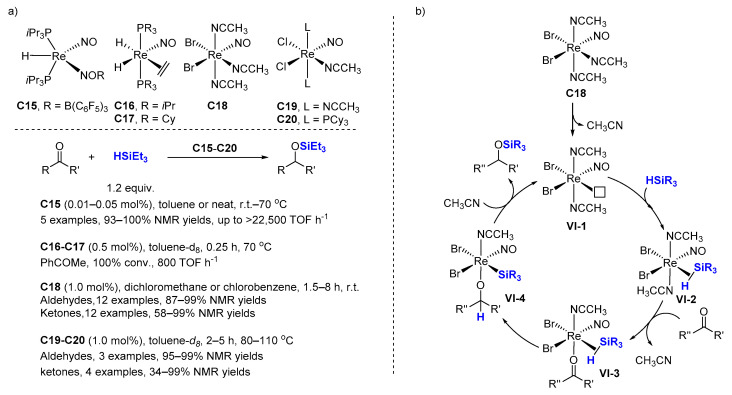
Rhenium oxides catalyzed hydrosilylation of carbonyl compounds. (**a**) Scope of the reaction; (**b**) Proposed mechanism.

Berke and co-workers reported several easily available nitrosyl-rhenium complexes, for example, Re(H)(P*i*Pr_3_)_2_(NO)(NOB(C_6_F_5_)_3_) (**C15**) [44] in 2005, Re(H)_2_(η^2^-C_2_H_4_)_3_(NO)(PR_3_)_2_ (**C16,** R = *i*Pr, **C17,** R = Cy) [45] in 2008, Re(CH_3_CN)_3_Br_2_(NO) (**C18**) [46] in 2009, Re(CH_3_CN)_3_Cl_2_(NO) (**C19**) [47] and Re(CH_3_CN)Cl_2_(NO)(PCy_3_)_2_ (**C20**) [47] in 2011. Those nitrosyl rhenium complexes were employed in the catalytic hydrosilylation of a variety of carbonyl compounds (Scheme 8a). Hydrosilylations of carbonyl compounds were carried out with the catalyst **C15**, HSiR_3_ (1 equiv., R = Et or Ph) from r.t. to 70 °C either in toluene or under solvent-free conditions. TONs up to 9000 and TOFs up to 22,500 h^−1^ were observed. **C16** and **C17** exhibited similar activity in hydrosilylation of acetophenone (0.5 mol% cat. loading at 70 °C in toluene-*d*_8_, giving full conv. and TOF up to 800 h^−1^).

For **C18**, chlorobenzene was found to be superior to all the other solvents used. Various aliphatic and aromatic silanes were tested. Excellent yields were achieved at r.t. in dichloromethane using triethylsilane, the reaction affording TOF values of up to 495 h^−1^.

Phosphine-free complex **C19** proved to be less effective than the bisphosphine derivative **C20**, as the reaction of benzophenone and Et_3_SiH (1.12 equiv.) with 1.0 mol% of **C19** was carried out at 80 °C, a conversion of less than 10% was achieved within 4 h, while in the same conditions, **C20** gave a 99% yield.

A possible mechanism for the hydrosilylation of ketones catalyzed by **C18** was proposed (Scheme 8b). The initial step consists of the dissociation of a CH_3_CN ligand to generate the pre-catalyst **VI-1** followed by the coordination of the silane to the rhenium center to form a η^2^–silane complex **VI-2**. Displacement of a second CH_3_CN ligand by the ketone at the rhenium center yields complex **VI-3**. The silyl-oxy complex **VI-4** could be obtained either from the transfer of a hydride from the silane to the ketone atom or from the oxidative addition of the silane followed by a β-hydride transfer process. Finally, the reductive elimination step produces the siloxy product and regenerates the pre-catalyst **VI-1**.

**Scheme 9:**
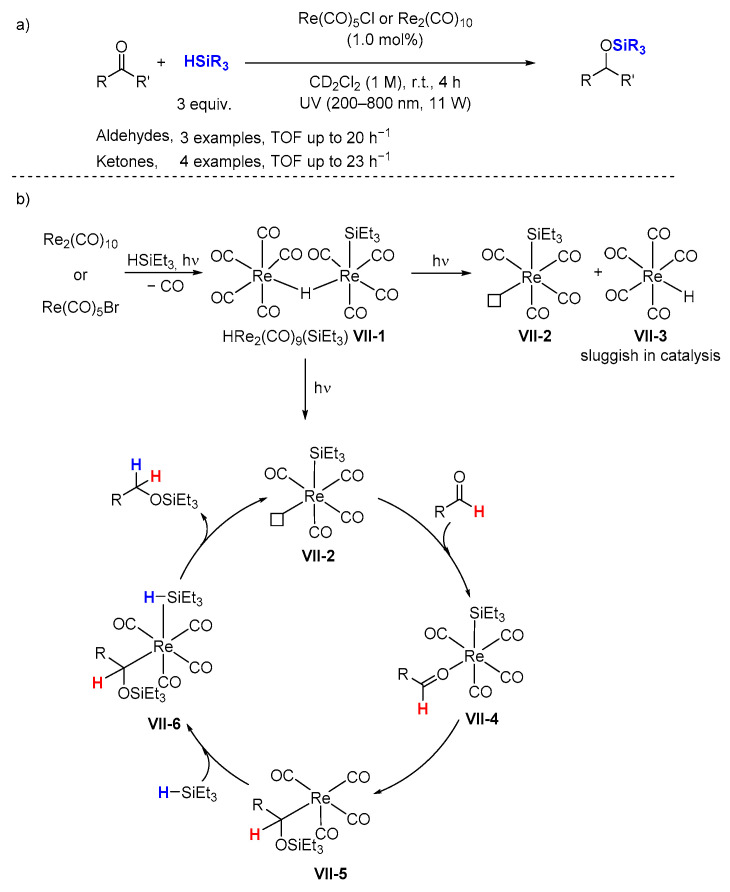
Hydrosilylation of carbonyls via Re(CO)_5_Cl photolysis (**a**) and proposed mechanism (**b**).

In 2012, carbonyl rhenium(I) complexes, namely Re(CO)_5_Cl (**C21**) and Re_2_(CO)_10_ (**C22**), have been found by Fan and co-workers [48] to be effective catalysts for the hydrosilylation of carbonyl substrates with various silanes and with TOF of 20−25 h^−1^ for aldehydes (Scheme 9a). In this methodology, 1.0 mol% catalyst and a Et_3_SiH:carbonyl ratio of 3:1 were used. When different silanes such as Ph_2_SiH_2_ and Ph_3_SiH were used, a decrease in the corresponding silyl ether yield was observed.

A detailed mechanism for the hydrosilylation of carbonyl compounds was proposed by the same author to account for the experimental observations (Scheme 9b). Upon photolysis of Et_3_SiH with Re(CO)_5_Cl or Re_2_(CO)_10_, a dimeric rhenium carbonyl species (**VII-1**) with a bridging hydride was identified in the mixture. When **VII-1** was isolated and tested for aldehyde hydrosilylation, the silyl ether was generated about 2–3 times faster in comparison to Re(CO)_5_Cl.

Upon photolysis, the dimer complex **VII-1** dissociates to afford Et_3_SiRe(CO)_4_ (**VII-2**) and HRe(CO)_5_ (**VII-3**) which was found to be inactive in catalysis. Then, the coordination of the carbonyl substrate to the vacant site of **VII-2** facilitates the silyl ligand shift onto the oxygen atom and forms an alkyl ligand bound to the Re center **VII-5**. Another silane coordinates to the Re center in **VII-5** via a η^2^-silyl complex or a σ-silyl (σ_H_) complex. Migration of a H atom from the silane to the alkyl group yields the silyl ether product and regenerates the catalyst **VII-2**. When either the carbonyl or silane has been depleted, the Et_3_SiRe(CO)_4_ complex(**VII-2**) coordinates back to HRe(CO)_5_ (**VII-3**) and becomes part of the resting state **VII-1**.

### 2.2. Hydrosilylation of Nitriles

**Scheme 10:**
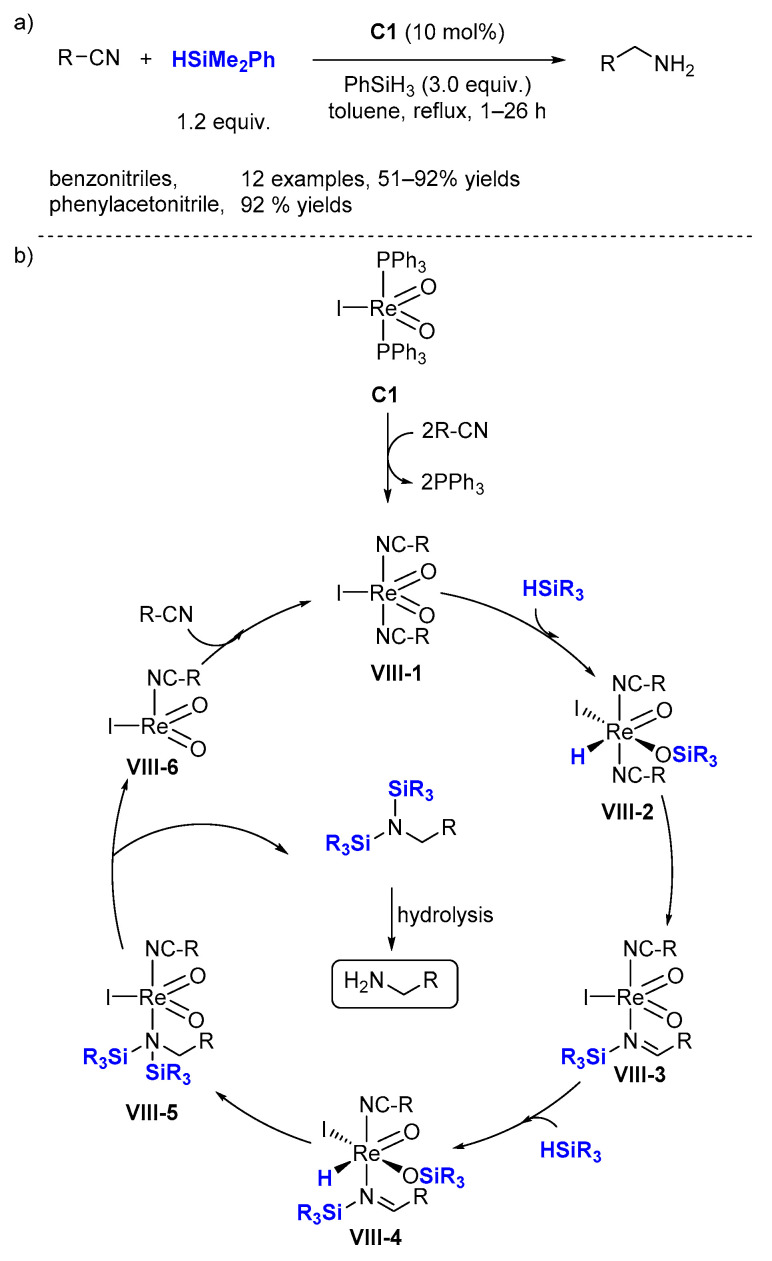
Reduction of nitriles with silanes and ReIO_2_(PPh_3_)_2_ (**a**) and proposed catalytic cycle (**b**).

The reduction of nitriles using rhenium are rare. However, in 2011, Fernandes reported a new catalytic system for the reduction of nitriles into the corresponding primary amines with silane as a reducing agent and catalyzed by oxo-rhenium complexes. Using (10 mol%) of rhenium(V)-dioxo complex **C1** and 3 equiv. of phenylsilane in refluxing toluene under air atmosphere, a large range of nitriles bearing functional groups, such as halogen derivatives, OCH_3_, SCH_3_, SO_2_CH_3_ and NHTs, was reduced with high chemoselectivity (Scheme 10a) [49].

Then, the catalytic cycle was proposed by the same author (Scheme 10b): Replacing two phosphine ligands with two nitriles at the rhenium center in **C1** produces complex ReIO_2_(nitrile)_2_ (**VIII-1**). The addition of the Si-H bond to one of the oxo-rhenium bond results in the formation of the hydride species (nitrile)_2_(O)IRe(H)OSiR_3_ (**VIII-2**). Dihydrosilylation of the nitrile to the corresponding *N*,*N*-disilylamino-rhenium complex **VIII-5**, followed by hydrolysis of the *N*-disilylamine, affords the primary amine as product.

### 2.3. Reductive Amination

**Scheme 11:**
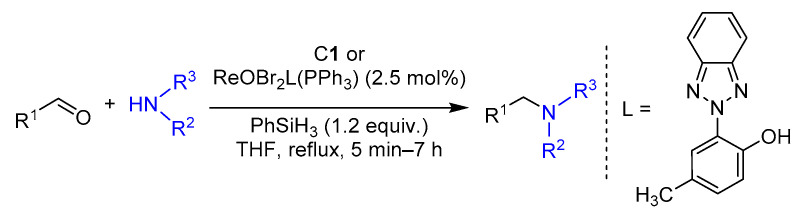
Direct reductive amination of aldehydes using silane/oxorhenium system.

The direct reductive amination of aldehydes with primary and secondary anilines [50], using the same oxorhenium complex **C1,** was achieved by the group of Fernandes. A large variety of functional groups, such as nitro, sulfone, ester, nitrile, amide, halides including iodide, were well tolerated, using 2.5% of catalyst loading under refluxing THF for 5 min to 7 h (Scheme 11) [51]. The same mechanism as in Scheme 10 was proposed by the authors. In 2013, the same group has reported a series of oxorhenium complexes containing heterocyclic ligands, which were found to also be very efficient catalysts for such transformation. For instance, similar efficiency and chemoselectivity were obtained with the oxo-rhenium complex [ReOBr_2_(L)(PPh_3_) **C23** (L = 2-(2′-hydroxy-5′-methylphenyl)benzotriazole) (Scheme 11) [52].

**Scheme 12:**
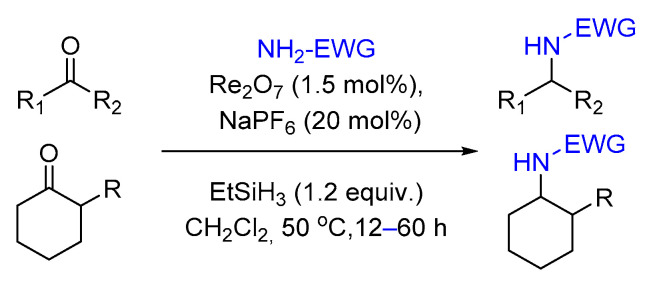
Direct reductive amination of ketones with electron-deficient amines using Re_2_O_7_/NaPF_6_ catalyst system.

Furthermore, in 2013, Ghorai reported a direct reductive amination of ketones such as alkanones and cycloalkanones with electron-deficient amines using Re_2_O_7_ (1.5 mol%) and NaPF_6_ (20 mol%) as the catalytic system and triethylsilane (1.2 equiv.) as the reductant in dichloromethane at 50 °C for 12–60 h (Scheme 12) [53]. Excellent diastereoselective reductive amination of 2-alkyl cyclohexanones was also observed, the formation of cis-selective 2-alkyl amines was obtained with high chemoselectivity (up to >1:99).

### 2.4. Hydrosilylation of Nitro Derivatives

**Scheme 13:**
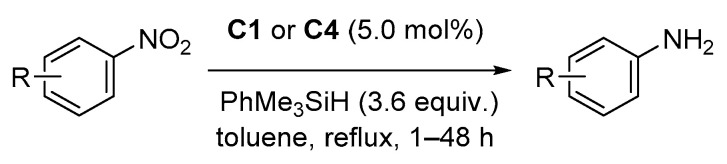
Reduction of aromatic nitro compounds with **C1** or **C4**.

In 2009, the group of Fernandes also reported the reduction of aromatic nitro compounds to the corresponding amines with silanes catalyzed by high valent oxo-rhenium complexes **C1** or **C4** in the presence of 3.6 equiv. of PhMe_2_SiH in refluxing toluene conditions for 1–48 h (Scheme 13) [54]. Aniline derivatives were then isolated in moderate to good yields (31–96%). It is noticeable that this catalytic transformation tolerated a huge variety of functional groups such as halides, esters, amides, sulfones and nitriles. By contrast, the hydrosilylation of nitroalkanes, such as 2-nitroethylbenzene, led to the corresponding nitrile in 38% yield.

### 2.5. Hydrosilylation of Carboxyl Acids Derivatives

**Scheme 14:**
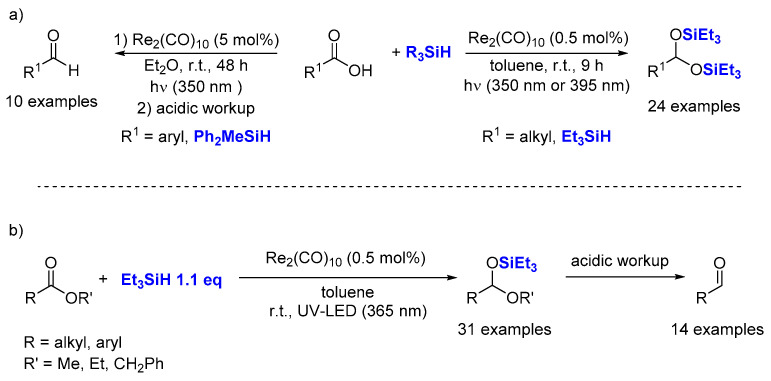
Rhenium-catalyzed chemoselective reduction of carboxylic acids (**a**) and esters (**b**) to aldehydes with hydrosilanes.

Following our study of the hydrosilylation of various carboxylic acids catalyzed with Mn_2_(CO)_10_ [55], our group recently reported the direct reduction of carboxylic acids [56] and esters [57] catalyzed by Re_2_(CO)_10_, under mild conditions (r.t., irradiation 350, 395, or 365 nm), with low catalyst loading (0.5 mol%) in the presence of a stoichiometric amount of Et_3_SiH as a reducing agent (Scheme 14). A large variety of carboxylic acids and esters was reduced in moderate to good yields to the corresponding protected aldehydes without noticeable formation of silylethers arising from over-reduction. Good functional group tolerance was achieved over amino, halogeno, isolated C=C as well as heteroaromatic groups. In addition, this new protocol was applied to a range of benzoic acid derivatives that are reluctant to be reduced by traditional methods with more drastic conditions: Re_2_(CO)_10_ (5 mol%), Ph_2_MeSiH (4.0 equiv.) to afford the corresponding aldehydes after acid treatment (Scheme 14a).

## 3. Hydrosilylation of C=C Bonds

### 3.1. Hydrosilylation of Alkenes

**Scheme 15:**
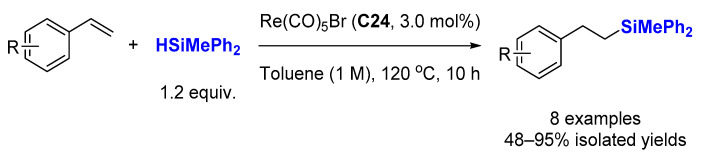
Re(CO)_5_Br-catalyzed hydrosilylation of styrenes.

Despite the importance of the hydrosilylation of alkenes for the production of silicones, only limited examples have been reported with rhenium catalysts in contrast to the hydro-elementation of polar bonds. In 2006, the group of Hua reported the addition of hydrosilanes to styrenes catalyzed by low-valent rhenium complexes, such as Re(CO)_5_Br (**C24**), to selectively afford anti-Markovnikov adducts in good to high yields (Scheme 15) [58]. For the hydrosilylation of styrenes, 1.2 equiv. of HSiMePh_2_ was employed at 120 °C in toluene (1 M) for 10 h. It is worth mentioning that other rhenium complexes, such as Re(CO)_5_Cl (**C21**) and Re_2_(CO)_10_ (**C22**), also showed catalytic activity for the hydrosilylation of styrenes, although less actively than **C24**, to give silylated alkanes in moderate yields as well as a slightly decreased selectivity. However, CpRe(CO)_3_ (**C25**) and NH_4_ReO_4_ (**C26**) did not show any catalytic activity at all in such a transformation. Aliphatic alkenes, such as 1-octene and methyl methacrylate, were reduced to their corresponding adducts in poor yields (30% and 28%, respectively).

**Scheme 16:**
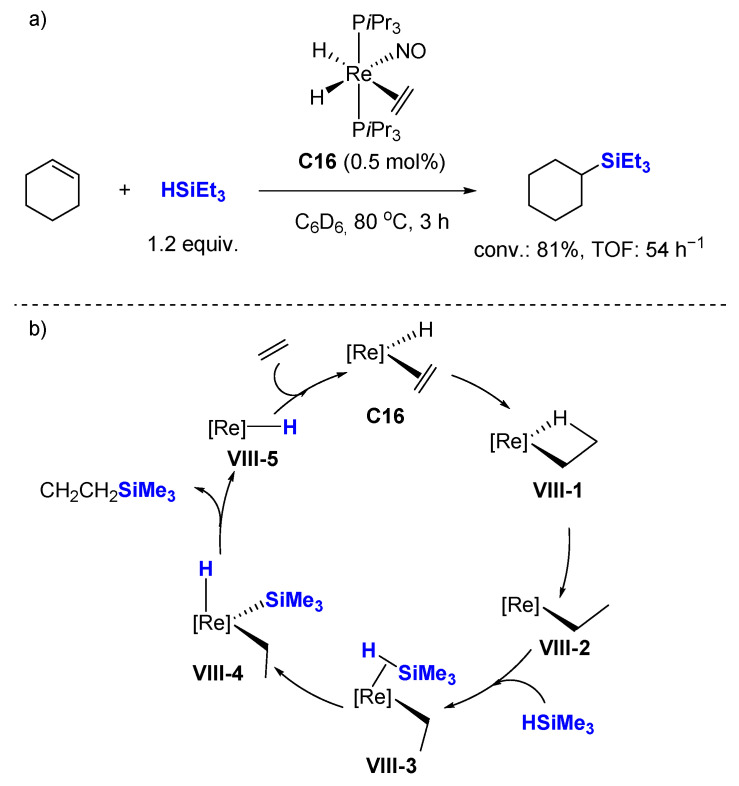
Rhenium mono-nitrosyl complex catalyzed hydrosilylation of alkenes (**a**) and proposed mechanism (**b**).

Interestingly, rhenium mono-nitrosyl complex **C16** developed by Berke et al., can also promote the catalytic hydrosilylation of cyclohexene [45] (Scheme 16a) under similar conditions that those employed for ketones (0.5 mol% cat., 80 °C, 3 h). This process was investigated theoretically by the group of Li with ethylene and trimethylsilane as model reactants (Scheme 16b) [59]. The proposed catalytic cycle starts with the insertion of the ethylene ligand into the Re-H bond, followed by the cleavage of the agostic interaction involved in **VIII-1** to afford **VIII-2**. Then, a silane molecule coordinates to the Re center to give a σ complex **VIII-3**. The next step involves an oxidative addition process to afford a seven coordinate complex **VIII-4**. Last step consists of a reductive elimination process to form the product. The catalytic cycle completes by coordination of an ethylene to **VIII-5**.

**Scheme 17:**
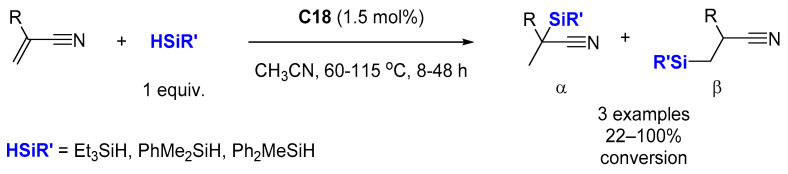
Re-catalyzed hydrosilylation of nitrile substituted olefins.

In 2014, the same group reported a highly selective hydrosilylation of acrylonitrile and its derivatives catalyzed by **C18** (1.5 mol%) by reaction with alkyl or aryl silanes (1 equiv.) at 60–115 °C in 8–48 h (Scheme 17) [60]. The regioselectivity of all the reactions were found to be around α:β = 3:1. Improved regioselectivity was obtained with a high ratio of acrylonitrile:silane.

### 3.2. Dehydrogenative Silylation of Alkenes

**Scheme 18:**
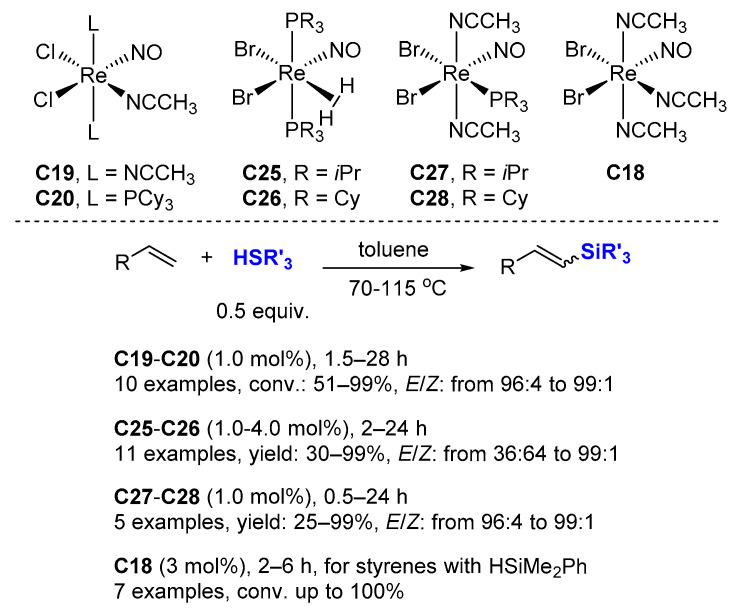
Rhenium mono-nitrosyl complexes catalyzed dehydrogenative silylation of alkenes.

Dehydrogenative silylation is often considered an undesired side reaction in the hydrosilylation of alkenes, but the selective production of vinylsilanes is also a valuable transformation. In this context; Berke et al. reported several rhenium mono-nitrosyl complexes (**C18** [60], **C19**–**C20** [47], **C25**–**C26** [61], **C27**–**C28** [62]) catalyzing the dehydrogenative silylation of alkenes with high chemoselectivity to produce silyl alkenes, along with the corresponding alkanes, with 1.0–4.0 mol% cat., at 70–110 °C in toluene-*d*_8_ (Scheme 18). Hydrosilylation products appear only rarely in very minor amounts. The complexes **C19**–**C20** and **C27**–**C28** gave better *E*/*Z* selectivity (96:4→99:1). Noticeably, ethylene can be transformed quantitatively into the corresponding dehydrogenative silylation product with **C27**–**C28** as catalysts with a ratio dehydro/hydro silylation products of 79:21 and 68:32, respectively. **C18** catalyzes the dehydrogenative coupling of styrene derivatives with HSiMe_2_Ph leading to dehydro/hydro silylation products in ratio of about 85/15.

## 4. Conclusions

In conclusion, we have highlighted in this review the diversity of rhenium complexes that can be encountered as pre-catalysts for hydrosilylation reactions. The oxidation state of the reported catalysts spans from +VII to +I leading to an extraordinary variety of coordination complexes, but also diverse mechanisms. It is now evident that rhenium can be an alternative to noble metals for hydrosilylation reactions, but we strongly believe that this less-studied metal also has great potential to discover new or complementary reactivity and selectivity in this field, in particular for alkyne derivatives and in other catalytic processes.
